# Tubeimuside I improves the efficacy of a therapeutic *Fusobacterium nucleatum* dendritic cell-based vaccine against colorectal cancer

**DOI:** 10.3389/fimmu.2023.1154818

**Published:** 2023-05-03

**Authors:** Yanan Tong, Guoxiu Lu, Zhiguo Wang, Shanhu Hao, Guoxu Zhang, Hongwu Sun

**Affiliations:** ^1^ Department of Nuclear Medicine, General Hospital of Northern Theater Command, Shenyang, China; ^2^ College of Medicine and Biological Information Engineering, Northeastern University, Shenyang, Liaoning, China; ^3^ Department of Microbiology and Biochemical Pharmacy, National Engineering Research Center of Immunological Products, College of Pharmacy, Third Military Medical University, Chongqing, China

**Keywords:** colorectal cancer, *F. nucleatum*, tubeimuside I, dendritic cells, vaccine

## Abstract

**Introduction:**

*Fusobacterium nucleatum* (*F. nucleatum*) infection has been confirmed to be associated with the development, chemoresistance, and immune evasion of colorectal cancer (CRC). The complex relationship between the microorganism, host cells, and the immune system throughout all stages of CRC progression, which makes the development of new therapeutic methods difficult.

**Methods:**

We developed a new dendritic cell (DC) vaccine to investigate the antitumor efficacy of CRC immunotherapy strategies. By mediating a specific mode of interaction between the bacteria, tumor, and host, we found a new plant-derived adjuvant, tubeimuside I (TBI), which simultaneously improved the DC vaccine efficacy and inhibited the *F*. *nucleatum* infection. Encapsulating TBI in a nanoemulsion greatly improved the drug efficacy and reduced the drug dosage and administration times.

**Results:**

The nanoemulsion encapsulated TBI DC vaccine exhibited an excellent antibacterial and antitumor effect and improved the survival rate of CRC mice by inhibiting tumor development and progression.

**Discussion:**

In this study, we provide a effective strategy for developing a DC-based vaccine against CRC and underlies the importance of further understanding the mechanism of CRC processes caused by *F. nucleatum*.

## Introduction

CRC is one of the most commonly diagnosed lethal cancer in the world and it is on the rise in young people. In recent years, this frequent and deadly neoplasm showed a significant increase in both the incidence and the mortality in Asia, especially in China (1, 2). Since Peyton Rous fiest revealed the causal relationship between microbes and cancers w in 1911 (3), various microorganisms have been found to promote the development of tumors. *Fusobacterium nucleatum* is a mutualist infectious agent and oncogenic microorganism that may contribute to colorectal carcinogenesis. *F. nucleatum* influences many stages of CRC progression, including bacterial enrichment, immune response and infiltration of myeloid cells, treatment and recurrence, and metastasis (4). Many noteworthy researches reveal that an abundance of *F. nucleatum* is highly correlated with poor prognosis and tumor recurrence in CRC patients, and the enrichment of *F. nucleatum* in CRC tissue is positively associated with high CRC specific mortality (5). In light of these results, a variety of therapeutic approaches were implemented to treat CRC with *F. nucleatum* infection. Compared with routine clinical therapies, immunotherapies (6), *F. nucleatum*-directed vaccines (7), and phage-based therapeutics (8) have been considered potentially more effective methods to treat CRC with *F. nucleatum* infection. However, due to the complex interactions between *F. nucleatum* and its host, the effectiveness of these treatments needs to be further confirmed. In contrast to some infectious diseases, typical control, elimination, and eradication efforts may not successfully prevent *F. nucleatum-*associated conditions, including CRC. We must uncover more about how *F. nucleatum* influences host cells and other microorganisms before considering *F. nucleatum*-targeted therapies (4). In our opinion, immune escape is one of the most important mechanisms by which *F. nucleatum* promotes cancer. First, *F. nucleatum* was found to inhibit the immune response of a variety of host cells to help them survive and proliferate in the cells, like epithelial cells, macrophages, tumor cells (9, 10). Second, *F. nucleatum* was also shown to shape the tumor microenvironment (TME) by influencing the accumulation of myeloid cells and blocking antitumoral immune responses of NK cells (6, 11). Furthermore, *F. nucleatum* infection promoted an increased risk of recurrence and chemoresistance by modulating autophagy by suppressing specific miRNAs in host cells (12). These studies all indicated a complex relationship between the microorganism, host cells, and the immune system throughout all stages of CRC progression, which makes the development of new therapeutic methods difficult. Therefore, we conjectured that if the immune escape caused by *F. nucleatum* infection could be reversed, it may be possible to effectively control the development of CRC or even cure it. In this study, we developed a novel DC vaccine that targeted both *F. nucleatum* and tumor cells to improve the immune microenvironment of CRC.

**Figure 1:**
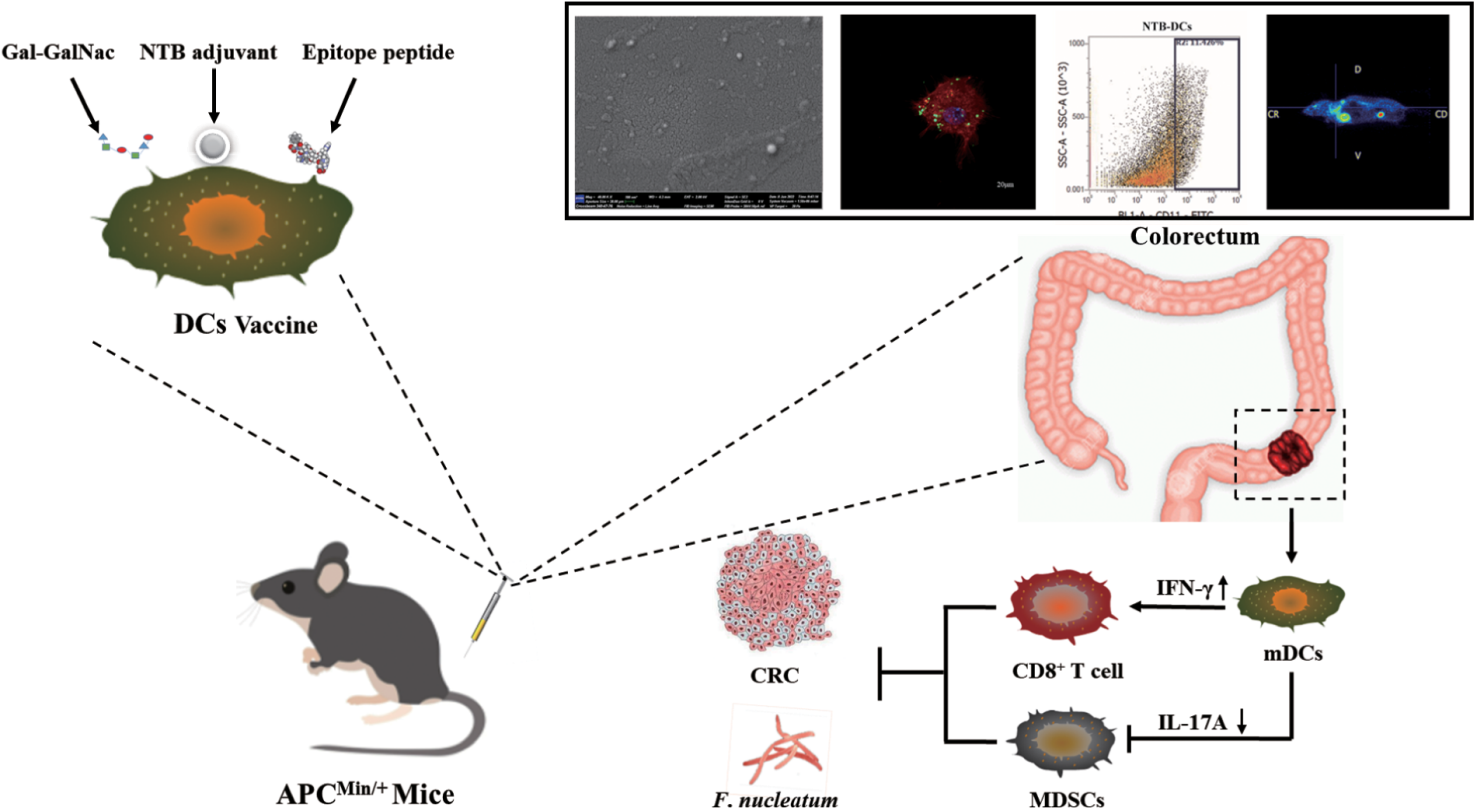
Schematic diagram of the therapeutic NTB dendritic cell-based vaccine against CRC.

DC is one of the most potent antigen-presenting cells (APCs) and can effectively activate natural and adaptive immunity by producing regulatory factors. DC vaccines are widely used in tumor immunotherapy because they can induce massive proliferation of T cells, consequently inducing long-lasting and powerful humoral and Cytotoxic T lymphocyte (CTL) immune responses (13, 14). To overcome the difficulty of intracellular infection of *F. nucleatum* and the potential of immune escape of CRC cells, we used immunogenic epitopes of FadA (15) and Fap2 (16) from *F. nucleatum* and a specific sugar residue, Gal-GalNAc from CRC tissues (17), to design the DC vaccine. Therapeutic vaccines, and particularly tumor neoantigen vaccines, usually require adjuvants to enhance vaccine potency because of the lack of effective activation of the immune system (18). Plant-derived adjuvants can strongly boost and direct immune responses and can be incorporated into different vaccine formulations. For example, *Pleurotus ferulae* polysaccharides acted as a potent adjuvant in a human papillomavirus DC vaccine to improve its antitumor efficacy (19), and astragalus polysaccharide was applied in tumor immunotherapy for modulating DCs (20). In our previous study, we screened a new powerful saponin adjuvant, tubeimuside-I (TBI), which simultaneously enhanced the vaccine immune response (21) and tumor suppression (22). In this study, we found that TBI simultaneously improved DC vaccine efficacy and inhibited *F. nucleatum* infection. In addition, we encapsulated TBI into nanoemulsion cavity (nanoemulsion-formulation- TBI, NTB) using a low-energy emulsification method due to the strong hemolytic toxicity of TBI. The results indicated that NTB significantly improved the humoral and cellular immune responses of *F. nucleatum* (*Fn)*-DCs compared with those observed in the control groups. Furthermore, compared with control groups, the NTB-*Fn*-DCs more effectively promoted antigen phagocytosis and the activation of APCs. Moreover, the NTB-*Fn*-DC vaccine significantly increased the activation status of CD8^+^ T cells and reduced the number of colonized *F*. *nucleatum* in the intestine. The number and volume of tumors in the intestine of a CRC APC^min/+^ mouse model were significantly decreased and long-term survival of mice with tumors was also greatly increased by the NTB-*Fn*-DCs vaccine. In conclusion, this research provides a potentially effective strategies to developa therapeutic DC-based vaccine against CRC and is also important for further understanding the mechanism of CRC processes influenced by *F*. *nucleatum* (**Figure 1**).

## Materials and methods

### Reagents

Gal-GalNAc (ZOM-TRI-017) was purchased from Omicron, USA. The sequences of Fap2 and FadA immunogenic epitopes were “TELAYKHYFGT” and “MKKFLLLAVLAVSASAFA”, respectively, and were composed by Abiocenter, China. The carcinogen azoxymethane (AOM, A5486) was purchased from Sigma, USA. Anaerobe basal broth (CM0957B) and anaerobe basal broth plates (CM0972B) were purchased from Oxoid, UK. RPMI 1640(11875101), DMEM (No Glu, 11966025), trypsin 0.25% solution (15050065), fetal bovine serum (FBS; 10099), and PBS buffer (10010031) were purchased from Gibco, USA. Murine MCSF (315-02) and interleukin (IL)-4 (214-14) were purchased from PEPROTECH. Goat anti-mouse IgG1 heavy chain (HRP) (ab97240), goat anti-mouse IgG2a heavy chain (HRP) (ab97245), and goat anti-mouse IgG2b heavy chain (HRP) (ab98703) antibodies were purchased from Abcam. A mouse IL-1β (432604), mouse IL-4 (431107), mouse IL-6 (431307), mouse IL-12p40 (431604), mouse IL-17A (432507), mouse IFN-γ (430807), and mouse TNF-α ELISA kits (430907) were purchased from Biolegend, USA. A mouse IFN-γ EliSpot kit (CT317-PR20) was purchased from Dakewe, China, and a mouse IL-17 ELISpot kit (A42927) was purchased from eBioscience. The anti-mouse-CD3, CD4, CD8, IFN-γ, CD11b, Ly-6C and Ly-6G fluorescent antibodies were purchased from BD Biosciences, CA, USA.

### Animals, bacterial strains, and ethics statement

Specific-pathogen-free male C57BL/6J-APC^Min/+^ mice (6 Weeks) were purchased from Huafukang Biotechnology Co. Ltd. (Beijing, China, animal license number: SCXK2020-0004). They were maintained at 26 ± 2°C, 50–60% humidity, and a 12 h light/dark periods in the animal room of the Pharmacy Department. *F. nucleatum subsp. Nucleatum* was kindly provided by Prof. Mao Xuhu (Third Military Medical University). All animal experiments were approved by the Experimental Animal Ethics Committee (Y (2021) 024).

### Preparation and characterization of NTB

The NTB adjuvant was prepared based on our previously described method. Chremophor-EL (Cremophor-35, BASF Co., Mumbai, India, cat^#^50043899) and Glycerol (Merck Co., USA, cat^#^G5516) at a 4:1 (w/w) ratio were mixed, and then oil phase Caprylic (Merck Co., USA, cat^#^C0426000)/Capric Triglyceride (Merck Co., USA, cat^#^T7517) at a ratio of 8:2 and TBI (100 μg/mL, HerbSubstance Co., Chengdu, China, cat^#^ PCS0035) were added. The NTB adjuvant is a clear and low viscosity homogeneous solution after equilibration. The TBI was replaced by water to prepare the blank nanoemulsion (BNE) adjuvant. The morphology of prepared NTB adjuvant was characterized by transmission electron microscopy (TEM; JEM-1230, Tokyo, Japan). The physicochemical characteristics of NTB were measured by Nano-ZS 90 (Malvern Instruments Ltd., Malvern, UK) and the size, polymer dispersity index (PdI), and zeta potential were recorded.

### Preparation of the *F. nucleatum* DC-based vaccine

The peripheral blood mononuclear cells (PBMC) were isolated from the femurs and tibias of APC^Min/+^ mice. Bone marrow-derived CD34^+^ progenitor cells were also collected from blood for backup. These cells were cultured in the presence of 20 ng/mL GM-CSF and IL-4 after density gradient centrifugation and magnetic-activated cell sorting (23). On day 7, the immature DCs were collected and stimulated with 20 μg/mL TBI, BNE and NTB adjuvant for 12 h, then 2 × 10^6^/ml of adjuvant-treated DCs were added with *F. nucleatum* Fap2 and FadA immunogenic epitopes (200 μg/mL of each peptide) and Gal-GalNAc (100 μg/mL). After 2 h, DCs were washed twice with phosphate-buffered saline (PBS) twice and then resuspended at a concentration of 5 × 10^5^ DCs/50 μL and designated as the TBI- *Fn*-DCs (TBI DCs), BNE-*Fn*-DCs (BNE DCs) and NTB-*Fn*-DCs (NTB DCs) vaccine and vaccine, and the PBS alone was used as a control (DCs Control).

### Treatment of the tumor model

We conducted animal experiments with two batches of mice. A batch of mice for survival rate observation experiment (n=10 each group) and the other batch of mice (n=20 each group) were used to take relevant experiment and samples, like: serum samples, spleens samples, PET/MR imaging and so on. Six-week-old C57BL/6J APC^Min/+^ mice (n=10) received antibiotics by gavage administration for 3 days and were given two cycles of one single intraperitoneal injection of AOM at a dose of 10 mg/kgfollowed by five successive days of 2% dextran sodium sulfate (DSS) in the drinking water (24). Then mice were administered *F. nucleatum* (10^8^ colony-forming units [CFU]) suspended in PBS (pH 7.4) via the intestinal tract every day for 20 weeks. Before the vaccine treatment, the mice were randomly grouping and immunized using a caudal vein injection twice a week with PBS, a 5 × 10^5^ DC Control, TBI DCs, BNE DCs, or NTB DC vaccine for 4 weeks. These mice were anesthetized with 40 mg/kg pentobarbital sodium and sacrificed for histopathological analyses after 24 weeks. Histological examination of the intestinal tissues was performed after hematoxylin and eosin (H&E) staining. The numbers of tumor from colorectal region were recorded.

### ELISA and ELISPOT

Blood sera were collected from all mice after vaccine treatment for 4 weeks. Serum samples were added to pre-coated wells of microtiter plates as primary antibodies. Detailed steps can be found in the latest reference (25) and the OD value of IgG subclasses antibody was measured at 450 nm.

The immature DCs were collected and stimulated with antigens (FadA, Fap2, and Gal-GalNAc) mixed with PBS, TBI, BNE, or NTB adjuvant for 24 h. The concentration of IL-1β, IL-6, and TNF-α cytokines in the supernatants were determined by ELISA kits. Splenocytes from immunized mice (n=10) were harvested and stimulated with antigen at a concentration of 2.5 × 10^6^ cells/mL in complete medium for 3 days. The levels of IL-4, IL-12p40, and IL-17A and IFN-γin supernatants were collected for cytokine assays.

The number of IFN-γ- and IL-17A-producing cells was evaluated by ELISPOT. The detailed steps were referred to our previous research. The spot-forming units were determined by ELISPOT Classic^®^ (AID, Germany) when the plates were dried.

### Flow cytometry analysis

The spleen lymphocytes were harvested as described above and stimulated with antigen at a concentration of 3× 10^6^ cells/mL in RPMI 1640 complete medium for 12 h. The proportion of cytotoxic T lymphocytes was detected by staining with CD3, CD4, CD8 and IFN-γ fluorescent antibodies.

Colorectal tissue was removed, opened and washed with Dulbecco’s Phosphate Buffered Saline (DPBS), without Calcium-Magnesium. Tumors were dissected, weighed, minced and incubated in Hank’s Balanced Salt Solution with 0.1 mg/ml collagenase D (Roche), 50 U/ml DNase I (Roche) and 50 µg/mg protease (Sigma-Aldrich) for 30 min at 37°C. Single cell suspensions were resuspended in cell staining solution and detected by staining with anti-mouse-CD11b, anti- Ly-6C and anti- Ly-6G fluorescent antibodies.

### PET/MR imaging

All mice fasted for more than 6 h prior to the detection of fasting blood glucose levels. ^18^F-FDG (18.5 ± 0.8 MBq, radiochemical purity more than 95%) was injected into the tail vein. After 50 min, mice were anesthetized by diethyl ether. The mice were fixed, and the integrated PET/MR equipment was used for synchronous image acquisition. The scanning time for MRI was 10 min. At the same time, PET image acquisition was performed, with a scan length of 20 min. MRI was used for attenuation correction, an AW post-processing system was used for image reconstruction, and the 3D region of interest (ROI) technique was used to calculate the ^18^F-FDG uptake rate per gram of all tissues (% ID/g).

### Determination of *F. nucleatum* abundance in mice colorectal tissue

A gentamicin protection assay was performed based on previous research with some modifications (26). In brief, the colon tissues were collected from different treatment groups and prepared for single cell suspensions. Next, 5×10^5^ cells/well were plated in 6-well sterile culture plates and incubated at 37°C under 5% CO_2_ for 12 h. Cells were washed twice with PBS and incubated with fresh culture medium containing gentamicin (100 μg/mL) for 1 h to kill extracellular and epithelial cell surface-bound bacteria. The cells were washed three times and lysed with 1 mL of 0.5% saponin (Sigma, USA) in PBS at 37°C for 15 min. Diluted cell lysates were plated on anaerobe basal broth plates (Oxoid, Basingstoke, Hants., UK) (27). Colonies were counted after 48 h. Experiments were performed at least three times in triplicate.

### Statistical analysis

For continuous data, comparisons between two groups were performed using the unpaired or paired Student’s *t* test or Mann–Whitney U test, where appropriate, and comparisons among multiple groups were performed using one-way analysis of variance. The log-rank (Mantel–Cox) test was used to compare the percent survival of the mice. All statistical analyses were conducted using GraphPad Prism 8 software (GraphPad Inc., San Diego, CA, USA). All values were expressed as the means ± SD, and significant differences were expressed as follows: *P < 0.05, **P < 0.01, and ***P < 0.001.

## Results

### Preparation and characterization of the NTB adjuvant

**Figure 2:**
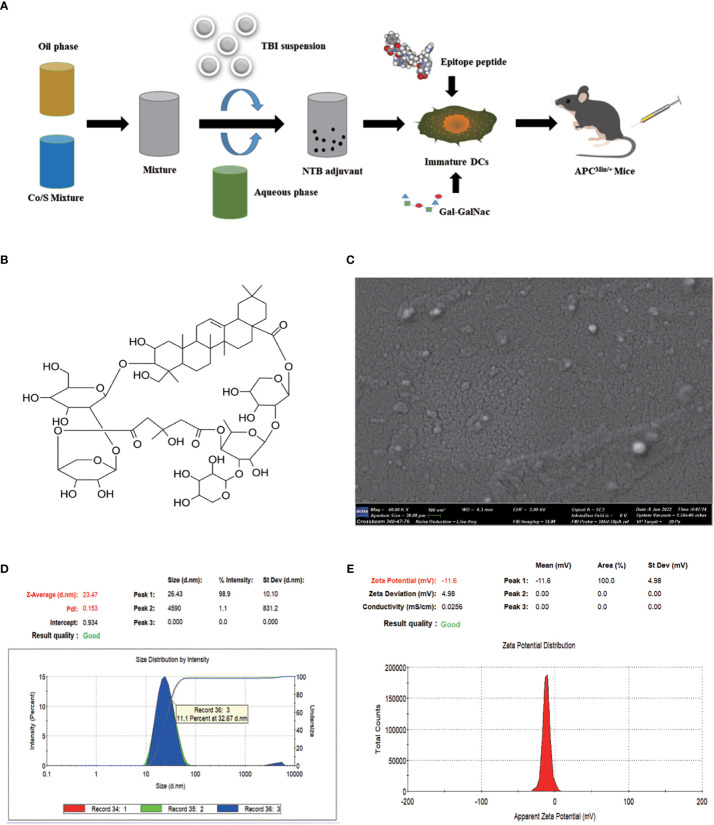
Preparation and characterization of nanoemulsion -TBI adjuvant. **(A)** Schematic illustration of NTB adjuvant and NTB- *Fusobacterium nucleatum* -dendritic cell-based vaccine preparation. **(B)** Structural formula of TBI. **(C)** Transmission electron microscopy picture of the NTB adjuvant. **(D, E)** Size distribution and zeta potential of NTB adjuvant.

As shown in **Figure 2A**, the nanoemulsion loaded with 100 μg/mL NTB was prepared by low-energy emulsification. TBI (**Figures 2B**, **S1**) is a natural saponin compound in *Bolbostemmatis rhizome*, a traditional Chinese herbal medicine. TBI is soluble in water, and TBI, BNE, and NTB adjuvants all presented a clear and transparent solution. The nanoemulsion droplets were white on dark background, which were mostly in the 1–100 nm range by TEM (**Figure 2C**).

The NTB droplets had a homogeneous particle size (23.47 nm, **Figure 2D**), good narrow distribution (PdI value was 0.153; the target PdI was < 0.3), and a stable zeta potential (−11.6 mV, **Figure 2E**). Moreover, after centrifugation at 13,000 g for 30 min, the NTB system exhibited a stable state without turbidity, phase or drug separation, creaming, precipitation, demulsification, or any other unstable appearance. In conclusion, the NTB adjuvant possessed a stable physicochemical property.

### The NTB adjuvant significantly promoted the activation of DC cells

**Figure 3:**
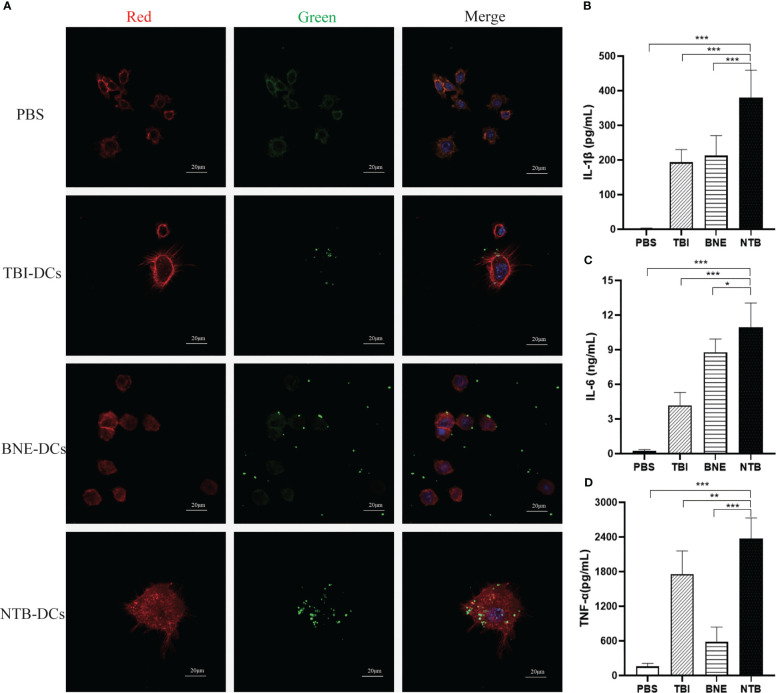
NTB adjuvant promote the immature DCs maturation and cytokines Secretion. **(A)** Antigen uptake of PBS, TBI, BNE and NTB formulated with GFP antigen by immature DCs. The DCs were imaged by CLSM after being incubated with naïve GFP protein for 30min. RED (Phalloidin-stained cell cytoskeleton), Green (GFP fluorescence) and Blue (DAPI-stained cell nucleus). Scale bar=20μm. **(B–D)** Effects of PBS, TBI, BNE and NTB-formulated antigen on the activation of immature DCs. The DCs were stimulated with PBS, TBI, BNE and NTB-formulated antigen for 24h, and supernatants were harvested for cytokine detection. Concentrations of cytokine (IL-1b, IL-6, and TNF-a) were detected by ELISA. The results are shown as the means ± SD. *P <0.05; **P < 0.01; ***P < 0.001.

DCs are the most powerful functional APCs and can absorb, process, and present antigens. “DCs provide an essential link between innate and adaptive immunity because they are capable of interacting with both B cells and T cells, thereby manipulating the humoral and cellular immune responses (28)”. Antigen uptake is a prerequisite for DCs activation, while TBI and NTB as adjuvants activate DC cells quickly and effectively. We applied Green fluorescent protein (GFP) rather than bacterial antigen for a more intuitive observation for examining the potentiation of antigen presentation. As shown in **Figure 3A**, there were a few GFP particles surrounded by phagosome-like vesicular structures in the TBI- and BNE-treated DC cells, while there was little green fluorescence observed in the PBS group. The green fluorescent dots scattered in the background indicated that TBI and BNE did not fully promote phagocytosis of DCs. By contrast, NTB-treated DCs engulfed all the GFP drops in the field of view and exhibited the strongest green fluorescence. These data suggested that the NTB adjuvant was more efficient than TBI or BNE for eliciting antigen capture by DCs.

The inflammatory cytokines play an important role in promoting DC cell maturation. As shown in **Figures 3B–D**, the secretion of IL-1β, IL-6, and TNF-α were significantly increased in TBI, BNE, and NTB groups compared with PBS group (*P* < 0.001). Moreover, NTB significantly increased the activation of APCs over that observed in the TBI and BNE groups (IL-1β, *P* < 0.001; IL-6, *P* < 0.05, TNF-α, *P* < 0.001). The levels of cytokine release induced in the TBI and BNE groups varied significantly within each group (*P* < 0.05). These results suggest that the NTB adjuvant effectively enhanced DC activation.

### The NTB DC vaccine enhanced the CRC model mice antibody response

**Figure 4:**
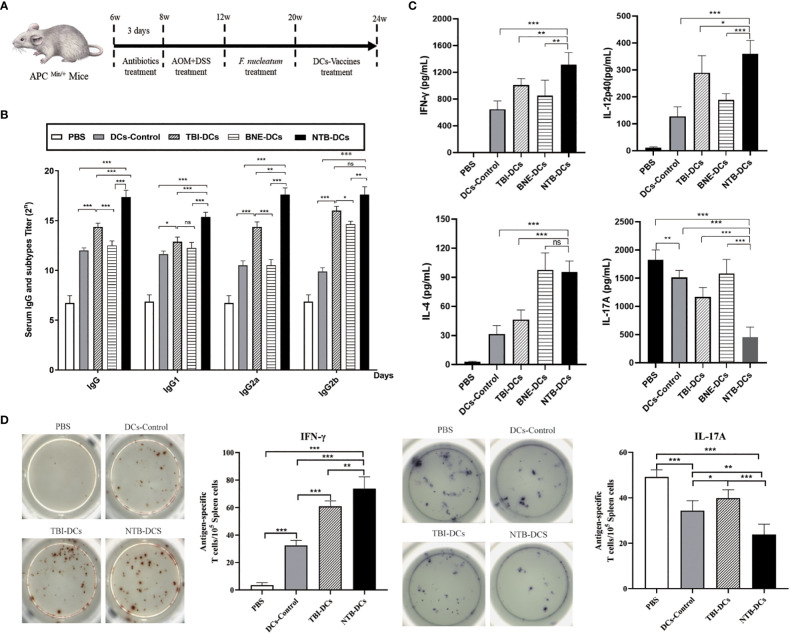
NTB-DCs vaccine significantly increased the antibody and cellular immune responses. **(A)** Six-week-old C57BL/6J APC^Min/+^ mice were given AOM for 4 weeks and administered *F*. *nucleatum* for 8 weeks. At week 20, the mice were immunized twice a week with DCs control, TBI-DCs, BNE-DCs vaccine and NTB-DCs vaccine for 4 weeks. **(B)** TBI and NTB adjuvant increased the antibody responses. Mice (n = 10) were immunized DCs control, TBI-DCs, BNE-DCs vaccine and NTB-DCs vaccine. At week 24, mice sera were harvested, IgG and IgG subgroups were detected by ELISAs. **(C)** TBI and NTB adjuvant significantly increased the cellular immune response. Splenocytes of immunized mice (n=10) were stimulated with antigen for 3 days. The levels of IFN-γ, interleukin-4 (IL-4), IL-12p40 and IL-17A in supernatant were determined using the corresponding ELISA kit. **(D)** ELISPOT analysis on IFN-γ and IL-17A spot-forming cells among splenocytes. The results are shown as the means ± SD. *P <0.05; **P < 0.01; ***P < 0.001.

Six-week-old C57BL/6J APC^Min/+^ mice were given AOM for 4 weeks, then administered *F. nucleatum* for 8 weeks and immunized with the DC control, TBI DC, BNE DC vaccine, or NTB DC vaccine for 4 weeks (**Figure 4A**). At week 24, sera were collected, and levels of antigen specific serum IgG and IgG subgroup antibodies were determined by ELISA. In **Figure 4B**, four DC vaccines all significantly increased titers of IgG than did the PBS group (*P* < 0.001). The TBI DC vaccine induced a higher IgG titer compared with the control and BNE DC vaccine (*P* < 0.01), indicating a potential adjuvant efficacy of TBI. In addition, the NTB vaccine induced the highest IgG titer compared with those induced by the DC control, TBI DC, and BNE DC vaccines (*P* < 0.001). IgG1 and IgG2a were used as markers for Th2 and Th1 responses, respectively (29). The IgG1 and IgG2a titer were used to explore the NTB vaccine-induced antibody responses to different Th polarizations. NTB vaccine induced a higher IgG1, IgG2a, and IgG2b titers than that in control and TBI groups (*P* < 0.01). Regarding the IgG2a/IgG1 ratio (**Figure S2**), the NTB vaccine had a higher value than did the control groups, suggesting that the NTB vaccine may induce a Th1-polarized immune response (30).

### The NTB DC vaccine enhanced multiple immune responses in the CRC model mice

Since the NTB DC vaccine induced Th1 immune response, we determined whether this adjuvant also resulted in a Th1-skewed cytokines profile. The levels of Th1 (IFN-γ, IL-12p40), Th2 (IL-4), and Th17 (IL-17A) cytokines in the supernatants of splenocytes harvested from vaccinated mice were measured by ELISA. As shown in **Figure 4C**, we found a significant increased level of IFN-γ and IL-12p40 in the TBI and NTB DC vaccine group compared with the DC control (*P* < 0.001), suggesting that the NTB vaccine enhanced the Th1 cytokine profile. Furthermore, the NTB vaccine induced a significantly higher Th2 cytokine IL-4 level compared with the DC control group (*P* < 0.001). The NTB vaccine also induced significantly decreased IL-17A production compared with the PBS and DC control (*P* < 0.001). This result is acceptable because IL-17 is a risk factor for CRC.

CTL monitoring by ELISPOT is a gold standard for the evaluation of antigen-specific T cell immunity in clinical trials and vaccine candidates. Cytokines can be used to distinguish different subsets of activated T cells by ELISpot. “For example, IFN-γ, IL-2, IL-6, IL-12, IL-21, and TNF-α are characteristic of T-helper (Th) 1-type cells; IL-4, IL-5, IL-10, and IL-13 of Th2 cells; and IL-17 of Th17 cells (31)”. The frequency of IFN-γ and IL-17A were evaluated to distinguish Th1 and Th17 cells. The NTB DC vaccine induced stronger Th1 (*P* < 0.001) responses compared with the DC control and significantly increased the relative ratio of IFN-γ-producing cells in splenocyte populations (**Figure 4D**). However, the IL-17 response was the opposite of IFN-γ, indicating that the IL-17-related immune responses were suppressed. This was similar to the results of the cytokine responses. In conclusion, the NTB DC vaccine induced strong Th1 and Th2 cellular responses.

### The NTB DC vaccine improved immune response and TME in CRC mice

**Figure 5:**
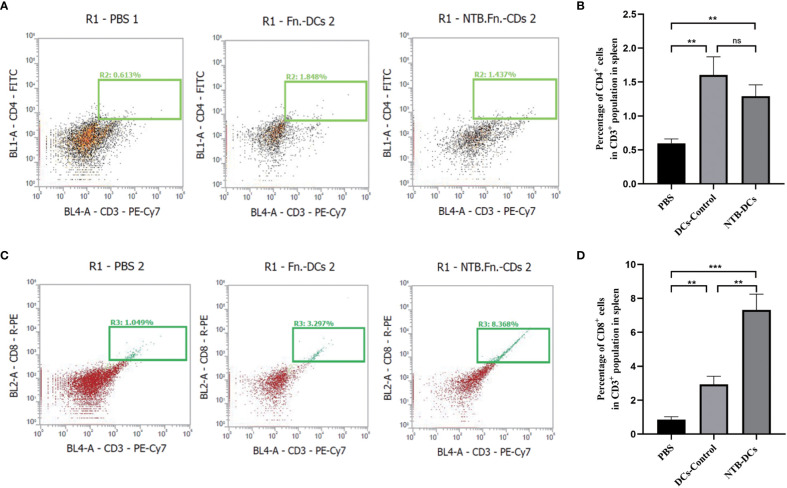
The NTB DC vaccine improved immune response in CRC mice. **(A, B)** Effects of the NTB vaccines and the proportion of CD4+T cells in the spleen. **(C, D)** Histogram of the percentage of CD8+T cells in the spleen. Mean percentages ± s.e.m are shown within each plot. n = 6. **P < 0.01; ***P < 0.001; ns, P>0.05.

The purpose of tumor immunotherapy is to induce tumor-specific effector T-cell responses, and it is also important to clear the existing *F. nucleatum* in the host cells. The activated T-cell responses can kill tumor cells and induce memory responses to prevent recurrence (23). We evaluated the proportions of effector CD3^+^CD4^+^ (**Figure 5A**) and CD3^+^CD8^+^ (**Figure 5C**) T cells in the spleen of mice treated with NTB DCs vaccine. As we can see, there is a certain amount of increase in the the proportion of CD4^+^ (**Figure 5B**, *P*<0.01) and CD8^+^ (**Figure 5D**, *P*<0.01) T cells in the DCs vaccine mice spleen compared to PBS treated mice. In addition, compared to the DCs Control, NTB vaccine increased a stronger proportion of CD8^+^ cells in the spleen (*P*< 0.01), while few differences in the proportion of CD4^+^ cells.

**Figure 6:**
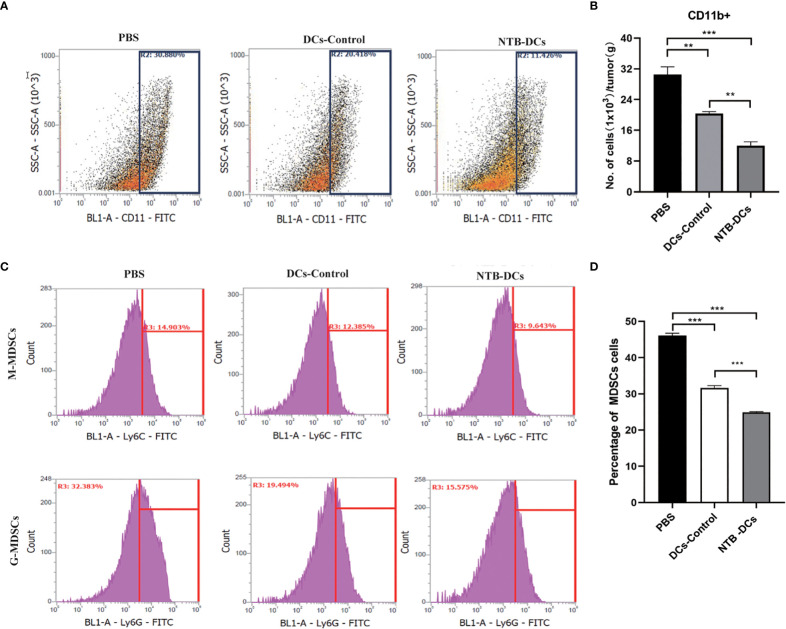
Intratumoral immune cell analyses after NTB-DC vaccine treatment. **(A)** Percentage with representative density plots of CD11b^+^myeloid cells. **(B)** The number of CD11b^+^ myeloid cells in tumors. **(C)** Representative histograms of monocytic MDSCs, CD11b^+^Ly6C^hi^Ly6G^-^, granulocytic MDSCs, CD11b^+^Ly6C^int^Ly6G^+^ and isotype control by flow cytometry. **(D)** Percentage of MDSC cells in different groups. *P <*0.05; **P < 0.01; ***P < 0.001.

“Myeloid-derived suppressor cells (MDSCs) are traditionally considered among the major components of TME (32)”. Some studies have shown that *F. nucleatum* selectively expands MDSCs to promote tumor progression (6). In our study, the decreased number of CD11b^+^ myeloid cells were observed in the tumors of DCs control and NTB vaccine groups (**Figure 6A**). The NTB DC vaccine could significantly inhibit the expansion of myeloid-derived immune cells caused by *F. nucleatum* (**Figure 6B**, *P*<0.001). “MDSCs mainly include monocytic MDSC (M-MDSCs, CD11b^+^Ly6C^hi^Ly6G^-^) and granulocytic MDSC (G-MDSCs, CD11b^+^Ly6C^int^Ly6G^+^) (33)”. So, we defined the different subsets of tumor infiltrating myeloid cells (**Figure 6C**), the numbers both of M-MDSCs and G-MDSCs were significantly decreased in NTB and DCs control groups, compared with PBS group (**Figure 6D**, *P*<0.001). These data further demonstrate that NTB DC vaccine plays a positive role in tumor progression and modulation of the antitumor immunity.

### The NTB DC vaccine significantly inhibited tumor progression and improved the survival rate of CRC mice

**Figure 7:**
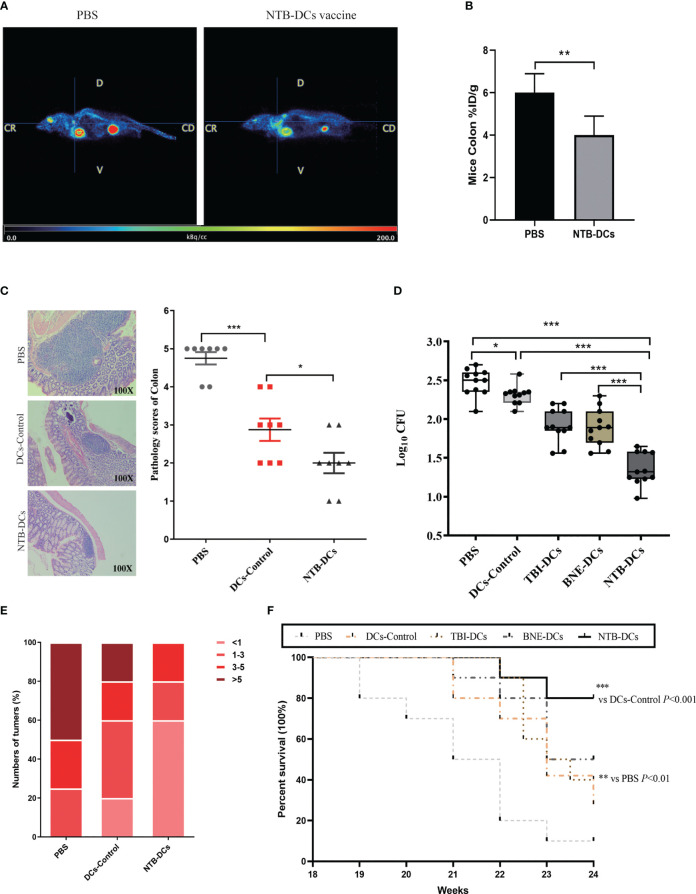
NTB-DCs vaccine significantly reduce the abundance of *F*. *nucleatum* in colon, numbers of CRC tumors and improved the survival rate of CRC mice. **(A)** PET/MR merge images of mice. **(B)** Comparison of ^18^F-FDG uptake rates in the ROI of mice colon after PBS and NTB-DCs treatment. (%ID/g: % injected dose per gram of ^18^F-FDG in the ROI.) **(C)** Intestinal tissue from CRC mice colorectum were collected and the representative histopathological sections are shown (magnification: x100). Severity scores of intestinal tissues were shown in the figure. **(D)** Diluted cell lysates from CRC mice colon tissues were plated on Anaerobe Basal Broth plates and colonies were counted after 48h. The *F*. *nucleatum* burdens in were measured. **(E)** The numbers of CRC from mice colorectum were counted after sacrifice. Mice are grouped according to the number of tumors and presented in the figure as a percentage. **(F)** The survival rates were monitored from 18 to 24 week (n=10 per group by log-rank [Mantel-Cox] test). The results are shown as the means ± SD. *P <0.05; **P < 0.01; ***P < 0.001.

After PET/MRI fusion imaging and 3D ROI reconstruction, the median sagittal section of mice in the PBS and NTB DC group was obtained, as shown in **Figure 7A**. Colors from left to right represent the high, medium, and low ^18^F-FDG uptake rates, corresponding to high, medium, and low levels of colon glucose metabolism. We were able to observe a significant increase in the uptake of ^18^F-FDG in the PBS-treated mice (**Figure 7B**, *P* < 0.01). The images indicated that the metabolism of the tumor tissue was significantly reduced after vaccine treatment. At week 24, we then sacrificed the mice and obtained their colorectal tissue for H&E staining. The NTB group exhibited fewer abscesses and less inflammatory cell infiltration than did the PBS and control groups. The histological analyses and severity scores demonstrated a minor colorectal tissue damage in the NTB group compared with the PBS group (**Figure 7C**, *P* < 0.001) and DC control group (*P* < 0.01). Due to the intracellular infection pattern of *F. nucleatum*, the cytotoxic immune responses were equally important for removing intracellularly infected bacteria. Therefore, we examined live bacteria in CRC cells to evaluate the clearance of *F. nucleatum* by the vaccine. The results were encouraging, as *F. nucleatum* could not continue the colonization in CRC tissues by means of immune escape due to the use of the DC vaccine. As shown in **Figure 7D**, there was little *F. nucleatum* colonization in the CRC tissue compared with the PBS group (*P* < 0.001), while the DC control (*P* < 0.05), TBI DC (*P* < 0.001), and BNE DC groups (*P* < 0.001) also exhibited an excellent antibacterial effect.

Subsequently, we measured the number of colorectal tumors in the different groups. More than half of the mice in the PBS group had more than five tumors; however, there were few cases in the NTB DC group (**Figure 7E**). The number of colorectal tumors in the DCs control mice was also significantly reduced compared with the PBS group (**Figure S3**). The survival rates in mice were determined until week 24. As shown in **Figure 7F**, groups that received DCs formulated with an adjuvant (TBI, BNE, or NTB) all had higher survival rates (40%, 50%, and 80%, respectively) than that of the PBS group (10%). Furthermore, the NTB DCs group induced the strongest protective effect compared with the other groups (*P* < 0.001). Therefore, we concluded that immunization with the NTB DC vaccine induced a highly protective effect in CRC mice and effectively stopped the tumorigenic process.

## Discussion

Vaccine development is often accompanied by an increased dependency on adjuvants to enhance vaccine potency because vaccine design has focused on pursuing higher safety with fewer side effects in the past decades. Therefore, the search for new adjuvants for vaccines is attracting increasing attention. DC vaccine therapy is one of the newest immunotherapy approaches to combat cancer, and it needs a powerful adjuvant to promote strong T cell responses (34). “An important mode of action for such adjuvants is to prolong antigen exposure to DCs and to induce their maturation. These mature DCs are extremely effective in the activation of antigen-specific T cells, which is a pre-requisite for induction of potent and long-lasting cellular immunity (35)”. Plant-derived immunopotentiators are gaining increasing attention because of their medicinal properties, such as immunomodulatory, antiviral, antimicrobial, antidiabetic, anticoagulant, and antitumor effects (36, 37). Several plant-derived adjuvants have been used to improve vaccine efficacy, such as QS-21 (38) and inulin (39). TB1 (C_63_H_98_O_29_, CAS No.102040-03-9) is a triterpenoid saponin and belongs to “cyclic bisdesmosides” due to its novel cyclic structures with a dicrotalic acid bridge (40) that was considered the active center for adjuvant activity (41). “It also has shown a strong inhibitory effect on lung cancer cells, nasopharyngeal carcinoma cells, liver cancer cells, kidney cancer cells, and tongue cancer cells (42, 43)”. These advantages make TBI suitable for the DC vaccine we designed. However, the toxic and irritation effects of TBI have limited its clinical application prospect. Therefore, drug delivery system have developed to improve the efficacy of TBI and reduce its toxic effects (44). Our blank nanoemulsion system demonstrated high selectivity, reduced toxicity, improved drug efficacy, and reduced drug dosage and administration times compared with TBI alone. Compared with the DC control and TBI DCs, NTB DCs induced more potent humoral and cellular immune responses. These results suggested that NTB is a promising and robust adjuvant platform for DC vaccines, and that the NTB DC vaccine exhibits a highly protective effect in CRC mice.

In contrast to other tumors, the *F. nucleatum*-induced TME is complex due to specific modes of interaction between bacteria, tumors, and hosts (45). First, T cells mediate the adaptive cellular immune response and are pivotal for inducing anti-tumor activity *in vivo* (46). For example, an increased number of IFN‐γ^+^ CD8^+^ tumor‐infiltrating lymphocytes in the TME inhibited the growth of CRC (47), while CD3^+^ T cells also showed an antitumor effect in colon tumor patients (48). It is worth noting that the results showed a low CD4 and CD8 counts in each group, mainly attributed to the specific modes of interaction between bacteria, tumors, and hosts. Actually, that CD4 and CD8 T-cell composition in both the spleen and tumor varied among models (49). In our results, the numbers of IFN‐γ^+^ and CD3^+^CD8^+^ T were increased in the NTB DC vaccine group, which indicated that the DC vaccine could improve the TME to inhibit CRC progression. Second, IL‐17 recruits bone MDSCs, which usually have an immunosuppressive role in the colon TME (50). *F. nucleatum* also has the potential to induce MDSCs infiltration in the TME to promote intestinal tumorigenesis. The decreased levels of IL-17A and the number of Th17 cells induced fewer MDSCs in the NTB group compared with the PBS group. In brief, the NTB DC vaccine exerted an antitumor effect by promoting immunotherapy in the TME. However, these types of immune cells are only what we verified based on the literature, and there are more anti-tumor mechanisms that need to be further investigated.

Another reason for developing novel treatments for CRC is the increasing drug resistance caused by *F. nucleatum. F. nucleatum* suppressed specific miRNAs involved in autophagy to inhibit the sensitivity of CRC cells to 5-FU and oxaliplatin (12). In addition, resistance to penicillin has been reported. These drug resistance issues make it difficult for current conventional clinical therapies to be effective in CRC with *F. nucleatum* infection. There are still a number of antibiotics, including metronidazole, clindamycin, and β-lactam antibiotics, to which most clinical isolates of *F. nucleatum* are sensitive(51). However, because of concerns about antibiotic resistance, implementing antibiotic intervention is problematic in numerous ways. For example, metronidazole treatment reduced tumor volumes in patient-derived xenograft models of CRC with an enrichment of *F. nucleatum.* However, metronidazole also killed other anaerobic bacteria that improve the responses to chemotherapy and immunotherapy. Consequently, we consider that the development of TBI and NTB DCs vaccines is meaningful, because these vaccines combine both antibacterial and antitumor activities. The application of DC vaccines was found to significantly improve the TME of CRC. Furthermore, the elevated cytotoxic immune responses effectively decreased the levels of intracellular *F. nucleatum* in CRC tissues. However, we did not test the sensitivity of CRC cells to chemotherapeutic agents after vaccine treatment, and we will continue to explore this in subsequent experiments.

There are some limitations in this study. First, we selected the epitope peptides of FadA and Fap2 according to the literature. The most suitable antigens for DC vaccines may be other epitopes of FadA and Fap2, or other proteins of *F. nucleatum*. This work will be performed after we have established a stable DC vaccine construction route. Second, the *ex vivo* antigen-loaded DC-based vaccine protocol is expensive, labor-intensive, and operationally complex (52). “The DC-targeted vaccine is targeting the antigens, DNA molecules, or drug molecules to the DCs through specific receptors expressed on the cell surface (53)”. The approach of an *in vivo* DC-targeted vaccine is simpler and more suitable for clinical operation, and we will focus more attention to developing such vaccines in subsequent research (54). Finally, the adjuvant mechanism of TBI is not yet clear, and we also need to explore the possibility that an optimal vaccine adjuvant requires a combination of adjuvants rather than a single adjuvant entity.

## Conclusions

In the current study, we found a new plant-derived adjuvant TBI, which effectively promoted antigen uptake and the activation of immature DC cells. Encapsulating TBI in a nanoemulsion greatly improved the drug efficacy and reduced the drug dosage and administration times. The NTB DC vaccine exhibited an excellent antibacterial and antitumor effects and improved the survival rate of CRC mice by inhibiting tumor progression. To summarize, the NTB adjuvant is a promising and robust adjuvant platform for DC vaccines.

## Data availability statement

The original contributions presented in the study are included in the article/**Supplementary Material**. Further inquiries can be directed to the corresponding authors.

## Ethics statement

The animal study was reviewed and approved by Animal Ethical and Experimental Committee of the General Hospital of Northern Theater Command.

## Author contributions

YT and GL contributed equally to this work. SH, GZ, and HS designed the experiments and wrote the manuscript. YT and GL carried out the experiments. ZW analyzed the experimental results. All authors contributed to the article and approved the submitted version.
