# Correction: Tubeimuside I improves the efficacy of a therapeutic *Fusobacterium nucleatum* dendritic cell-based vaccine against colorectal cancer

**DOI:** 10.3389/fimmu.2025.1639717

**Published:** 2025-06-19

**Authors:** Yanan Tong, Guoxiu Lu, Zhiguo Wang, Shanhu Hao, Guoxu Zhang, Hongwu Sun

**Affiliations:** ^1^ Department of Nuclear Medicine, General Hospital of Northern Theater Command, Shenyang, China; ^2^ College of Medicine and Biological Information Engineering, Northeastern University, Shenyang, Liaoning, China; ^3^ Department of Microbiology and Biochemical Pharmacy, National Engineering Research Center of Immunological Products, College of Pharmacy, Third Military Medical University, Chongqing, China

**Keywords:** colorectal cancer, *F. nucleatum*, tubeimuside I, dendritic cells, vaccine

Author Guoxiu Lu was erroneously assigned to Affiliation 1 Department of Nuclear Medicine, General Hospital of Northern Theater Command, Shenyang, China. This affiliation has now been removed for author Guoxiu Lu.

The authors apologize for this error and state that this does not change the scientific conclusions of the article in any way. The original article has been updated.

