# Supplementary material for: Tubeimuside I improves the efficacy of a therapeutic Fusobacterium nucleatum dendritic cell-based vaccine against colorectal cancer
Source: Front Immunol. 2023 May 3;14:1154818. doi: 10.3389/fimmu.2023.1154818 (PMC10189021; doi:10.3389/fimmu.2023.1154818)
Supplement: Supplementary file 1 [file DataSheet_1.docx]

Supplementary Material

Tubeimuside I improves the efficacy of a therapeutic *Fusobacterium nucleatum* dendritic cell-based vaccine against colorectal cancer

**Yanan Tong^1^ ^†^, Guoxiu Lu^1,3^ ^†^, Zhiguo Wang^1^, Guoxu Zhang^1^*, Hongwu Sun^2^***

^1^Department of Nuclear Medicine, General Hospital of Northern Theater Command, No.83, Wenhua Road, Shenyang 110016, China

^2^Department of Microbiology and Biochemical Pharmacy, National Engineering Research Center of Immunological Products,College of Pharmacy, Third Military Medical University, Chongqing, China

^3^College of Medicine and Biological Information Engineering, Northeastern University, Shenyang, Liaoning, 110169, China

* Correspondence:

Guoxu Zhang*: zhangguoxu_502@163.com

Hongwu Sun*: sunhongwu2001@163.com

†These authors share first authorship：Yanan Tong and Guoxiu Lu contributed equally to this work.

Supplementary Figures


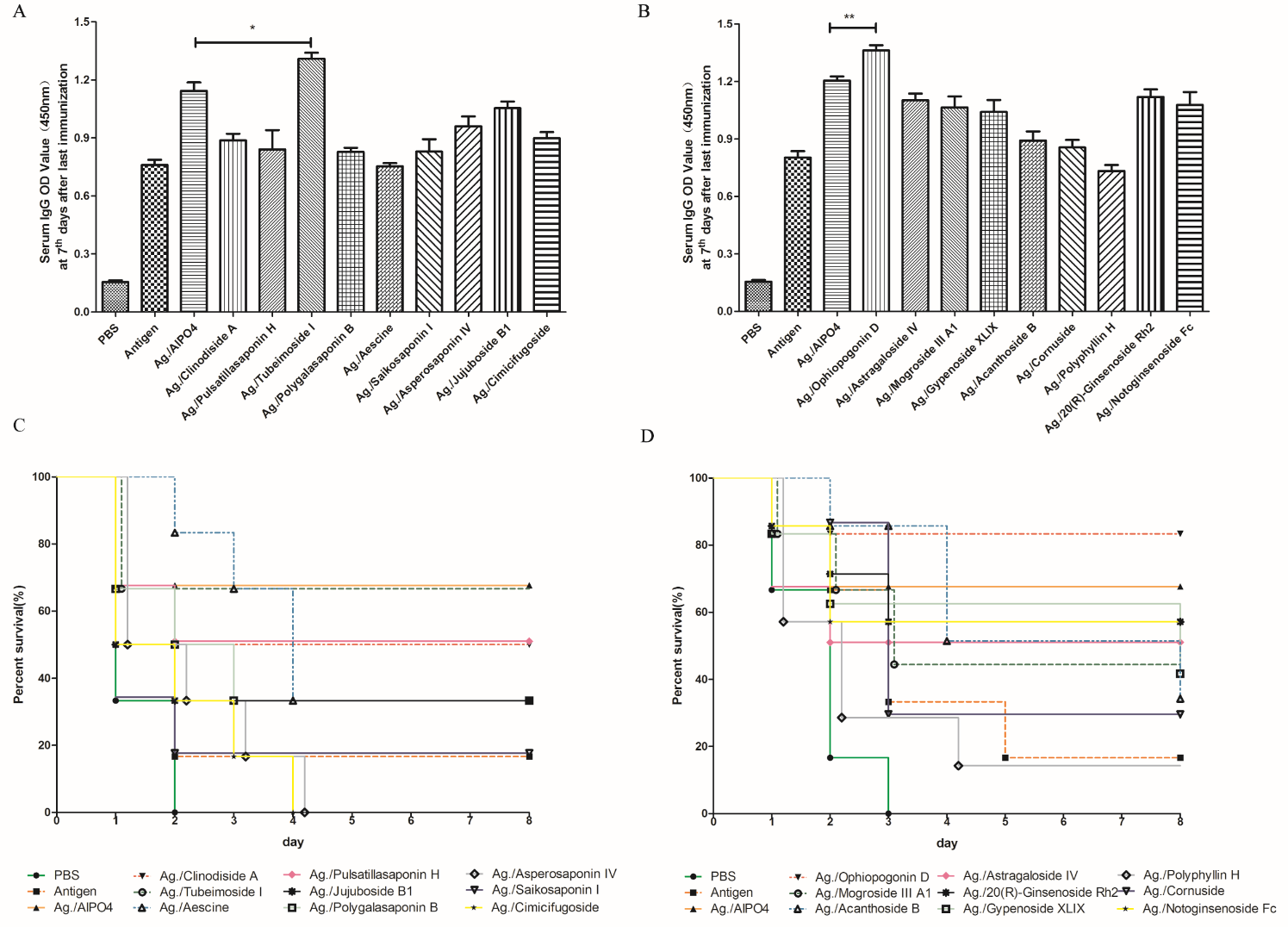


**Supplementary Figure 1.** In our previous study, we evaluated the adjuvant efficacy of 18 plant-derived compounds. Ophiopogonin D and Tubeimuside I induced a robust IgG antibody and high survival rates compared with Ag/AlPO_4_.(A-B) Absorbance (450nm) of IgG in mice surum at 7^th^ days after last immunization. (C-D) Mice (n = 10) were intravenously infected with MRSA 252 (1x10^9^ CFUs), and the survival rates were monitored for 14 days.


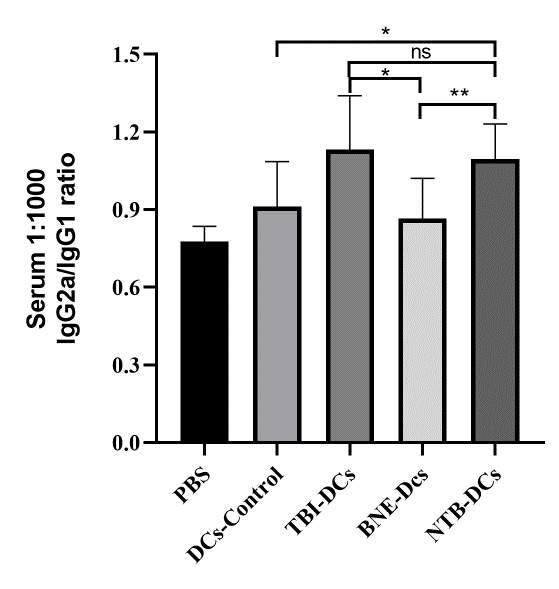


**Supplementary Figure 2.** The IgG2a/IgG1 ratio (optical density value at 450 nm) was calculated.


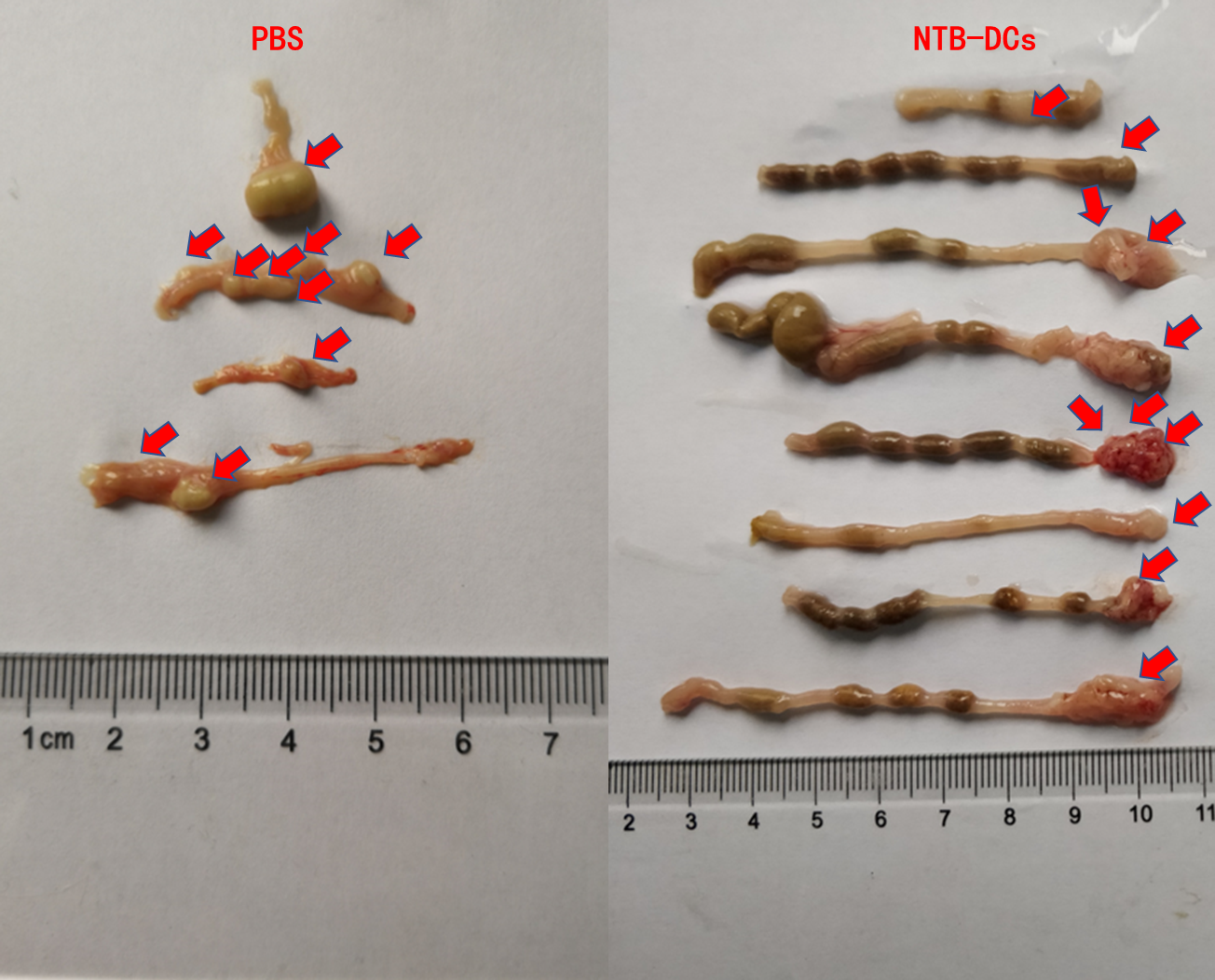


**Supplementary Figure 3.** The colorectum of mice is removed from the abdominal cavity and the tumor numbers were counted in PBS and NTB-DCs groups. The left part shows the colorectum tissues from PBS mice and the right part is the NTB group. Each red arrow represents a tumor.


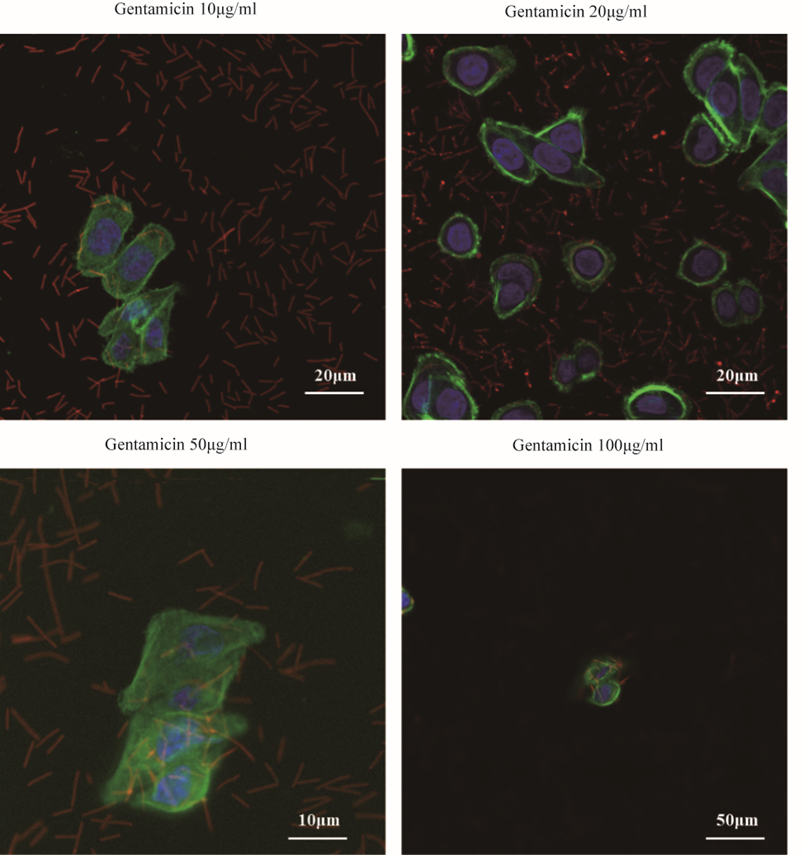


**Supplementary Figure 4.**  Gentamicin protection assay at different gentamicin concentrations. The *Fusobacterium nucleatum* were labeled with red fluorescence, the LoVo cells were labeled with green fluorescence and the nucleus were labeled with blue fluorescence.


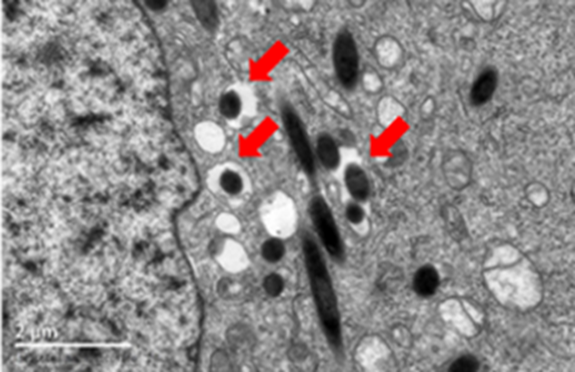


**Supplementary Figure 5.**  The intracellular F.nucleatum observed by TEM.


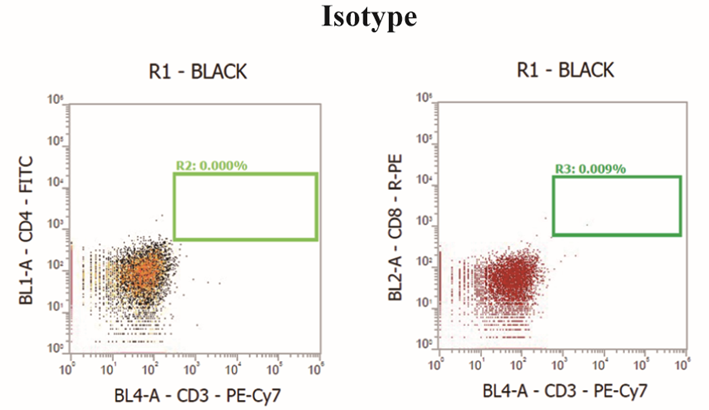


**Supplementary Figure 6.**  The controls of CD4+ and CD8+ T cells.


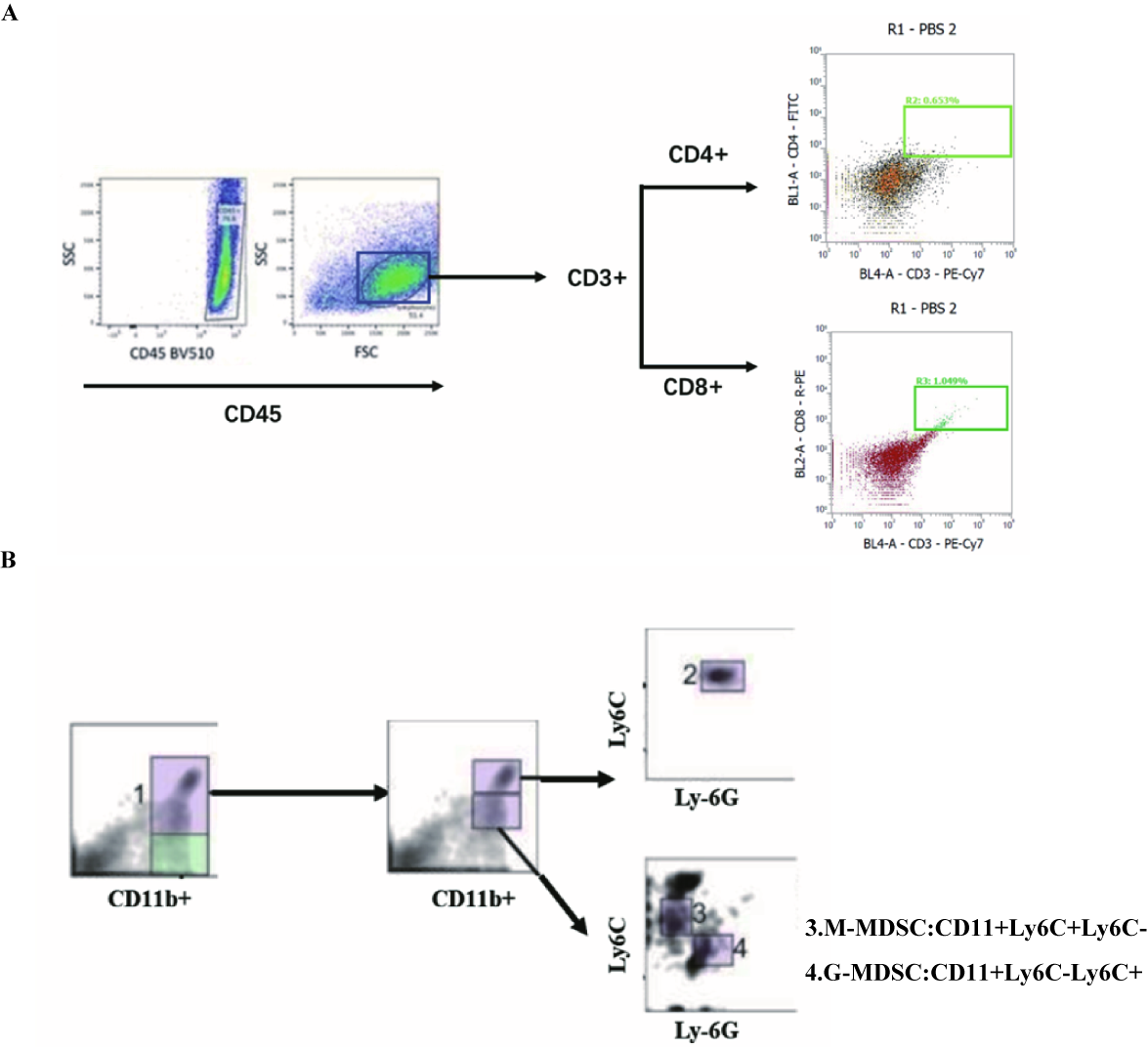


**Supplementary Figure 7.**  Schematic of the gating strategy.
