# Supplementary figures and images for: Tubeimuside I improves the efficacy of a therapeutic Fusobacterium nucleatum dendritic cell-based vaccine against colorectal cancer
Source: Front Immunol. 2023 May 3;14:1154818. doi: 10.3389/fimmu.2023.1154818 (PMC10189021; doi:10.3389/fimmu.2023.1154818)

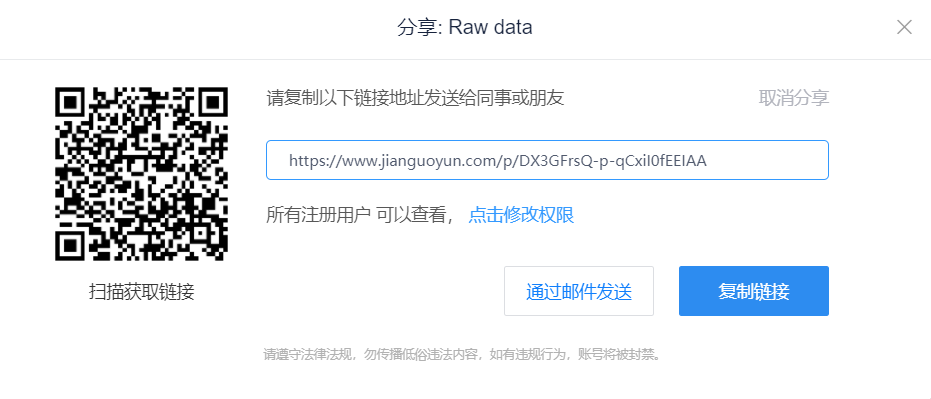

Supplement: Supplementary file 2 [file Image_1.tiff]
